# Memoir on a New Method of Cutting for the Stone

**Published:** 1836-07

**Authors:** 


### Art. IV.

*Mémoire sur une Manière nouvelle de pratiquer l'Opération de la Pierre.* Par le Baron Dupuytren. *Terminé et publié par* L. J. Sanson, Chirurgien de l'Hôtel Dieu, &c., *et par* L. J. Begin, Chirurgien en chef à l'Hôpital Militaire de Strasbourg, &c. *Avec dix Planches.—Paris,* 1836. Grand Folio. Pp. 50.

*Memoir on a new Method of Cutting for the Stone.* By Baron Dupuytren. *Completed and published by* J. L. Sanson *and* J. L. Begin. *With ten Plates.*—Grand Folio. *Paris and London,* 1836.

There is no bodily affliction to which the attention of the surgeon has, during all ages, been so constantly directed as stone in the bladder; and there is no operation which has been so variously performed, or upon which so much skill and inventive faculty have been brought to bear, as that for its removal. From the period of Celsus, who described the operation of lithotomy as it was performed by Ammonius of Alexandria and Meges at Rome, down to the present time, there will hardly be found a surgeon or a medical writer of any celebrity who has not devoted some of his pages to this subject. Instruments have been heaped upon instruments, varying in number and complexity, from the simple knife employed in the older times for cutting on the gripe, to the chest of tools which accompanied the surgeon who extracted the stone according to what was called the "apparatus major;" a name which doubtless became associated with the operation in consequence of the multiplicity of instruments made use of in the performance. The bladder has been successively assailed from all sides, and attacked at every point which offered the remotest chance of penetrating to the interior. Cautious timidity, doubtless the offspring of immature knowledge and reckless temerity, the result of utter ignorance, seem by turns to have directed the hand of the surgeon.

At one time the seat of mischief was approached with all the slowness and foresight of a wary general, desirous of securing the fortress with the least possible injury to its wall: at another, it was stormed with violence, and carried by a *coup de main*, which, while it succeeded in dislodging the enemy, involved the ruin of half the citadel which had contained him.

It is rather a curious fact, that the particular mode of operation which forms the subject of the work now before us should be almost identical with that described and recommended by Celsus, nearly two thousand years ago. The Baron Dupuytren, in a short memoir on the history of Lithotomy, declares his "new method" to be nothing more than a revival of that which was first promulgated at Alexandria and Rome, somewhat modified indeed by the result of his experience, and reduced to scientific principles by a more accurate knowledge of anatomy.

The Essay, to the consideration of which the succeeding pages will be devoted, was never completed by the Baron, although in the press at the time of his death. An extract from his will, which appears in the title-page, tells us that he appointed MM. Sanson and Begin to bring it before the public. It is therefore the more valuable, as being the last of his literary labours, as containing his ultimate opinions, and embodying the results derived from the experience of a whole life. We hasten, therefore, to lay before our readers a condensed view of the memoir, using, as far as our limits will permit us, the author's own words in all the more important passages.

The attention of M. Dupuytren had long been directed to the operation of lithotomy. Dissatisfied with the large average of deaths which followed the removal of the stone according to the ordinary methods, he applied himself earnestly to the task of investigating the causes of so many fatal results; of comparing the relative advantages embraced by the various means which had been resorted to from time to time for the extraction of calculus, as well as the objection to which each was liable; and of ascertaining whether a safer, a surer, and more expeditious mode of entering the bladder might not be adopted; one that would supersede the lateral operation, and the corresponding section of the prostate gland by the knife or single-bladed *lithotome caché*, the instruments usually employed in France for that purpose. We shall pass over his observations on these subjects, which include an elaborate and very beautiful anatomical essay on the parts situated between the bladder and perineum, illustrated by superb engravings, in order that we may at once proceed to his description of the "*Méthode nouvelle*," which was the result of his investigations and experience.

"To incise the integuments, and cut through the superficial muscular and aponeurotic layers down to the urethra; to open the canal to a sufficient extent; to make a lateral division of the neck of the bladder and the prostate gland on either side: these constitute the three principal steps of the new operation, which it was our object to render as easy, as certain, and as safe as is compatible with the existing degree of perfection in the surgical art. To accomplish this, it was necessary to review the instruments usually employed, and to introduce some important modifications of them. Our attention was first directed to the staff, the use of which is to render the canal of the urethra solid, conspicuous, slightly projecting, and easy to enter. The staffs in ordinary use, being too strongly curved and too much prolonged beyond the curve, were found not to correspond exactly with the canal they were destined to traverse and to occupy.* Their deficiency in bulk, and the smallness of the groove, rendered it difficult to feel them with precision through the walls of the urethra and the soft textures which cover the canal. The consequence of these defects has been, that even skilful surgeons have occasionally found themselves embarrassed in the attempt to discover and open the urethra, or that the knife has missed the groove of the staff, by slipping over its side; or, again, that, after its introduction, it has quitted the canal, and passed into the surrounding structures.

"The staff which we have adopted is made of wrought steel, and presents a curve rather more decided than that of the common 'algalies.' It is furnished with an ebony handle, the surfaces of which are rendered flat and rough, in order that it may not slip through the fingers of the assistant whose business it is to hold it. Towards the middle of the instrument, or just in the centre of the curve, it presents an enlargement, continued along the extent of about two inches; and this enables it to fill accurately the urethra. The groove is large and deep, with its edges rounded, turned outwards, and as it were everted; so that it may be easily recognized by the touch, even through a considerable thickness of soft parts. The extremity of this instrument is rounded, and terminates in an olive-shaped beak, which enables it to slide smoothly through the urethra, separating the parietes of the canal by unfolding them, and thus opening a passage to the expanded portion which is to follow. Lastly, the groove is gradually reduced in size, until it disappears altogether towards the extremity of the staff: this is done to obviate an inconvenience which has sometimes accompanied the use of the common instrument,—viz. the arrest and retention of the lithotome at the sudden termination of the groove, so as to render its extrication difficult.

"There is no doubt that a convex bistoury, with a firm blade, will sufficiently answer the purpose of dividing all the external parts and opening the urethra. We found it, however, more convenient to employ a double-edged knife, rounded towards the point, and with the blade fixed into the handle. Such an instrument makes a neater section of the tissues, is firmer in the hand of the surgeon, and likewise enables him (when the point is once planted in the groove of the staff,) to divide the urethra backwards and forwards, without removing it for the purpose of reversing the direction of the cutting edge.

"It cannot be denied that the *lithotome caché* of Frère Côme, or the

* It may be necessary to observe, that the common French staffs are much more curved and prolonged towards the extremity than those used for the same purpose in England. Even the reduction which M. Dupuytren has effected in this respect does not bring the instrument within the English standard, as may be seen by the plate which accompanies the memoir.

*bistouri boutonné,* are either of them competent to effect the division of the neck of the bladder and the prostate gland on both sides; but for this purpose they require a double introduction and a double manipulation, which not only prolongs the operation, but precludes the possibility of ensuring a symmetrical form to the entire incision, or giving it an equal extent on each side. A double lithotome is capable of obviating these important objections, because the two blades, separating from a common stalk and expanding in the bladder, cannot fail (on its withdrawal,) to make, through the neck of the bladder and prostate gland, a double incision, the precise limits of which may be accurately defined." (P. 23.)

The instrument made use of by the Baron in his latter operations was the double lithotome, invented, or rather improved upon, by M. Charriére. A very minute and technical description of it is given in the text, which we may condense by observing that it consists of two long narrow blades, folding upon each other, and concealed in a case which is slightly curved, and adapted, by its size and shape, to be passed along the groove of the staff into the bladder. Thus, the instrument is introduced through the urethra without injury to the parts, while a mechanical contrivance attached to the handle allows the blades to be expanded after it has been lodged in the bladder: they quit the sheath on either side, and, when separated, resemble the blades of a pair of scissors with the cutting edges reversed. In this state the instrument is withdrawn, and cuts its way out. The size of the opening produced, of course depends upon the extent to which the blades have been expanded, their degree of separation being indicated by an index.

*Description of the Operation.* "The patient should be placed in the common position adopted for performing the lateral operation. At one period we imagined that the surgeon might retain the staff in the bladder with the left hand, while with his right he cut into the perineum; but this method, which perhaps is feasible in the operation of Frère Jaques and Frère Côme, is not in the present instance to be recommended, as the left hand becomes necessary for the purpose of rendering the soft parts tense, and the left fore-finger must be used as a guide down to the groove of the staff. After the presence and probable size of the stone has been ascertained by the staff, the surgeon should give to the latter a vertical direction; so that the straight portion of the instrument may form a right angle with the axis of the body, while the curve should be kept somewhat elevated towards the symphysis pubis, rather than pressed downwards and backwards upon the rectum. A dextrous and trustworthy assistant should retain it precisely in this position. The surgeon then, with a double-edged knife, makes a semilunar incision across the perineum, cutting through the raphe about six lines anterior to the anus, towards which aperture the concavity of the wound is directed. The skin, the subcutaneous, elastic, cellular tissue, the superficial perineal aponeuroses, and the connexion between the sphincter muscle and the posterior extremity of the bulb, should be successively divided to the same extent as the external incision, until the staff and its groove can be distinctly felt. During this part of the operation, the mind should never lose sight of the direction of the urethra, and its relative position with regard to the intestine. The knife should be carefully kept away from the anterior bulging surface of the gut, and should be made to pursue the direction of an imaginary line, extending from the anus towards the anterior surface of the bladder and the hypogastrium. In more than one instance, while operating on the dead subject, the knife, from being directed too much backwards, has passed into the posterior region of the urethro-anal triangle, and pierced the rectum, instead of entering the urinary canal.

"The lower wall of the urethra should then be entered with the point of a knife, having a double-edged blade fixed in the handle; so that, by an easy motion backwards and forwards, the groove of the staff may be laid bare to the extent of about three or four lines. One important remark presents itself with respect to this incision: it is, that the point of the instrument should be carefully retained in the groove, in order to preclude even the possibility of its passing backwards, and denuding or perhaps piercing the rectum; the surface of which, at the apex of the triangle, comes nearly in contact with the prostate and urethra.

"The nail on the fore-finger of the left hand, which has been retained in the wound, should now be introduced into the groove of the staff, where it serves as a guide for the lithotome, the blunt extremity of which passes with ease through the incision. The convexity of its curve should be directed backwards towards the rectum, so that, while the concave surface is applied to the staff, and accommodates itself to the direction of the parts, it may the more readily be slided onwards into the bladder. The well-known sensation produced by the immediate contact of two metallic bodies informs us that the lithotome is well placed: the surgeon then seizes the staff with his left hand, raising it towards the pubes, and pushing its extremity more deeply into the bladder; while, at the same time, he introduces the lithotome along its groove.

"The staff should be withdrawn as soon as the flow of urine from between the instruments, and the striking of the calculus, indicate that this second step in the operation has been accomplished. The lithotome is then turned round, so as to direct its concavity backwards, and, after first using it as a sound for measuring the size and ascertaining the situation of the stone, the surgeon expands and slowly withdraws it from the bladder, at the same time gradually depressing the handle towards the anus, until the blades are completely disengaged from the wound. The instrument is thus made to pass over the projection of the rectum, and the possibility of the extremities of the blades approaching too near the walls of the intestine is altogether done away with.

"As soon as the lithotome has been withdrawn, the fore-finger of the left hand should be introduced into the bladder, for the purpose of measuring the extent of the incision, of ascertaining the condition of the parts, and, lastly, that it may serve as a guide for the forceps. The finger should bear against the posterior wall of the incision, in order to prevent the possibility of the forceps being passed between the bladder and rectum; which accident has been known to occur.

"We should find a difficulty in expressing how greatly the subsequent steps of the operation, for the purpose of discovering, seizing, and withdrawing the stone, become simplified and facilitated by adopting this method. If the foreign body prove soft and friable, the short and capacious passage allows us to wash out the bladder with copious injections of water, and thus to get rid of even the very smallest fragment that had been retained. It offers similar advantages in those not very uncommon cases where, from the presence of several calculi, it becomes necessary to reintroduce the forceps, and to repeat the manipulations for the extractions of the stones again and again.

"The details into which we have thus entered may be considered as affording sufficient evidence of the superior claims which this new method of operating possesses over that which has hitherto received the majority of favorable opinions." (P. 25.)

We shall pass over a short historical record, in which the author shows that he has but resuscitated and modified the old operation of Celsus, in order that we may proceed to the enumeration of the advantages which, in the opinion of M. Dupuytren, are to be derived from the bilateral section.

"1st. It is easier and quicker than most of the other methods, as well as equally safe.

"2d. The incision is made in the largest part of the lower outlet of the pelvis; a circumstance of such paramount importance in all operations for stone, that we may fairly estimate the comparative merit of different methods by taking into consideration the distance of the incision from that point; the operation being more or less eligible as it is commenced at a greater or less distance from the symphysis of the pubes.

"3d. It opens a more direct road from the surface of the perineum to the cavity of the bladder than any of the other methods, and very much facilitates the introduction and the manipulation of the instruments, the extraction of the stone, and the flowing away of the urine.

"4th. It is also better adapted for making an opening proportionate to the volume of the stone, and for extracting it without violence, dragging, distension, or laceration of the parts: it is therefore calculated to diminish the probability of subsequent inflammation in the neck or body of the bladder, the peritoneum, the kidneys, the cellular texture, &c.

"5th. The prostate gland can never be completely divided, however freely we may find it necessary to carry the incision through its substance.

"6th and 7th. While it affords the means of making a very large opening, the ejaculatory ducts are secured from all liability to injury. At the same time it enables us to avoid the large vessels, and prevent the hemorrhage which so often supervenes on the lateral operation.

"8th. Lastly, it is applicable to all ages, to both sexes, and to calculi of every size." (P. 28.)

In this memoir we find two statistical tables; the one showing the result of 356 lithotomy operations, performed in Paris and its environs during ten years; the other exhibiting the issue of eighty-nine cases in which the new method, or bilateral section, was made use of. In the former table the number of deaths are sixty-one, or rather more than one in six; the latter presents a mortality of nineteen, or one in four and two-thirds.

Were these tables to constitute our criterion for comparing the relative merit of the two operations, the "new method" would present any thing but a cheering prospect, and is certainly not much calculated to raise our opinion in its favour. It would not, however, be quite fair to submit it definitively to this test, more especially as the eighty-nine operations referred to were performed by various surgeons, all of whom might not be equally skilful, and consequently not equally successful in practising the bilateral section. But we cannot forbear expressing our disappointment, when we find that the advantages, which are so prominently brought forward and so highly extolled in the memoir, fall so short of our expectations, become so feebly maintained, or, it would be more accurate to say, are so palpably contradicted by the result of practical experience, as far as this has yet gone.

It is by no means our intention to throw down the gauntlet before the shade of this distinguished surgeon. We are convinced that our professional brethren will receive with gratitude the legacy bequeathed to them by so great a master; and those even, who, by a long course of practice in the operation for stone, may have fully satisfied themselves as to the best and most efficient method of opening the bladder, will pause, ere they reject as wholly inadmissible the evidence of such high authority. We might justly incur the reproach of national prejudice, of a deficiency in good taste and good feeling, did we attempt to impugn the experience of a long and active life; and it is with feelings of the most profound respect, combined with no small degree of diffidence, that we venture to criticise the opinions to which that experience has given birth. In the foregoing pages we have abstained from introducing any remarks of our own, convinced that we should best consult both the interest and the inclination of our readers by laying before them a condensed outline of the Baron's work, comprising all that is important as regards the operation itself and the advantages which it promises.

We were the more inclined to adopt this course, in the first place that we might do ample justice to the author, and again because the splendid and expensive manner in which the book is got up must effectually prevent it from enjoying a very extensive circulation. We may now, however, be allowed to make a few observations on a subject of so much importance, and perhaps to enter into a brief enquiry:

1st, as to the causes which render the ratio of mortality after lithotomy heavier in France than in this country; 2d, respecting the lateral operation itself, as it is performed by ourselves and by our French neighbours; and 3dly, whether the causes of death are diminished by the "Nouvelle Methode," or bilateral section.

At the commencement of the memoir, M. Dupuytren deplores the heavy average of deaths, which, according to his own statement, consists of one in every five or six patients who undergo the operation. It is to be regretted that the medical literature and records of our own country furnish us with very few similar tables whose accuracy can be depended upon: but we have no hesitation in stating our conviction that the sum-total of cases operated upon in England during the last twenty years would present a rate of mortality less by one-third than that exhibited in the French report. According to the information which we have been able to collect on the subject, deduced partly from the registers of public institutions, but principally from enquiries among the most experienced lithotomists, the average number of deaths may be fixed at one in eight.

Mr. Crosse, in his late work "On the Urinary Calculus," has furnished us with the account of 704 cases which had been cut at the Norwich Hospital during a period of above sixty years. These present an average mortality of one in seven and about three-fifths; and we are inclined to believe that the estimates of the distinguished surgeons who have operated there during the last twenty or thirty years, as well as those of most of the other skilful and successful lithotomists of this country, would display an average of success rather above than below the standard which we have fixed. We again repeat our regret for the scanty information which our statistical reports afford on this subject, a subject of the more importance, because, as it would appear to us that a pretty broad line of distinction exists between the methods employed in France and England for the removal of calculus, it becomes a matter of the greatest interest to ascertain with the utmost accuracy the result of the two modes of treatment in a large mass of cases.

Dr. Marcet, in his "Essay on Calculous Disorders," gives a statement of 506 lithotomy cases which occurred in the Norwich Hospital between the years 1772 and 1816. Of these, 70 died or 1 in $7\frac{1}{4}$. The result of operations since that period, of which there appear to have been 198, has reduced the total amount to 93 in 704, making an average of 1 in $7\frac{1}{2}$, and shewing an increase of success during the last twenty years; since of these 198 cases only 23 died, or 1 in $8\frac{1}{2}$.

In 529 out of the total 704 cases, as recorded by Mr. Crosse, the calculus did not exceed ℥i. in weight, and in most instances was considerably less: 47 of these died, making an average of 1 in $11\frac{1}{4}$.

Chesselden, during a period of twenty years, cut 213 patients, and lost 20, or 1 in $10\frac{5}{8}$.

We endeavoured to obtain a more complete result of the average mortality, by comparing the tables which Prout, Marcet, Smith, and some others, have taken great pains to collect and furnish on this subject; but the numerous discrepancies which existed in the different statements proved that there must have been some inaccuracy in the source from which they were derived.

According to Dr. Prout, the average number of deaths which occurred in the Leeds Infirmary after lithotomy, between the years 1767 and 1817, was 1 in $7\frac{4}{5}$. In the Bristol Infirmary 1 in $4\frac{1}{4}$.

The mean ratio of mortality in the Bristol, Leeds, and Norwich Hospitals, he estimates at 1 in 7¾.

Assuming, however, that the advantage is on our own side, we will venture to ask whether this advantage does not result from two causes. In the first place from a more judicious selection of cases, in which we can hope to confer benefits by removing the stone; and, again, by a superiority in our method of performing the operation. It not very unfrequently happens that stone is accompanied by other complaints, which preclude the possibility of a successful issue after its removal, more particularly in the adult subject, where renal disorder, or enlargement and disease of the prostate gland, give rise to the cachectic state, the gradual failing of the powers of life, to general irritation and a train of symptoms, which are apt to be wholly ascribed to the presence of the calculus, and which are therefore expected to subside after its extraction. The organic alterations and functional derangement of the kidneys, with their widely spreading effects on the general system, have been hitherto but little noticed among the French; and we have every reason to believe that many cases, in which we should consider the prospect of the operation as hopeless, and which are susceptible only of palliative measures to render the remainder of life as little wretched as possible, would there be at once condemned to the knife, on the principle that a foreign body existed in the bladder which must be got rid of *coute qui coute.* Indeed, we have reason to doubt whether the previous condition of the patient, the means of preparing him for the operation, or the subsequent treatment, are so seriously weighed and attended to, or so well understood in France as in this country.

We shall presently offer a few remarks on the detail of the operation itself; and we shall now only suggest, in general terms, whether the greater mortality in France may not in some measure be attributed to the exertion of an undue degree of violence for the extraction of the stone; a violence, from the practice of which we are perhaps not wholly exempt, but which certainly is more reprobated and discouraged here by our best lithotomists than is the case among our neighbours. We will ask whether the safety of the patient has not been sacrificed to the *éclat* of the operation; whether a saving of a few seconds of time in the performance is not occasionally sought for, and gained at the expense of a bruised prostate, a laceration of the neighbouring cellular connexions, and the almost inevitably fatal consequences which ensue thereon? A little less feeling of personal vanity, and we must add a little more attention to the dictates of humanity; a little less desire to secure the applause of the multitude assembled, delighted to see the stone conjured from the bladder into the hands of the operator, yet scarcely understanding how the process was effected; and a little more care and foresight directed towards the ultimate success of the operation, might possibly be more frequently rewarded by the consciousness of lives positively saved by it, as well as by the gratitude of those who had been restored from disease and misery to a state of health and vigour.

We feel the less hesitation in making the foregoing observations, as they involve a general principle which we shall always be proud to cherish and maintain, while we would disclaim in the strongest manner their personal application to the illustrious individual, whose very work now before us affords the most direct evidence how anxious he was to render the result of his immense experience subservient to the cause of humanity, and with what readiness he employed his splendid abilities to alleviate the sufferings of his fellow-creatures.

In an early part of the memoir, Mr. Dupuytren has enumerated the causes which lead to a fatal termination after the operation of lithotomy; they are shortly these: Nearly two-thirds of those who perish after the operation die from the effect of inflammation attacking the bladder, the cellular texture of the pelvis, the rectum, the small intestines even as far as the stomach, the peritoneum, the kidneys, the pleura, the lungs, and the liver. These latter organs being situated at a distance from the seat of operation, cannot of course become affected from continuity of texture, but are probably predisposed to disease, and, under the general irritation of the system, take on an inflammatory action. About a fourth of the fatal cases die of hemorrhage and its consequences, while the small residue sink under various concomitant and accidental disorders. These inflammations of the bladder, pelvic fascia, and neighbouring viscera, all seem to have their origin from the immediate vicinity of the incision by which the bladder was entered.

Are they not all referable to one cause, viz. the extreme narrowness and want of room at the part where we seek to divide the prostate and neck of the bladder? This space is so straitened, that we are unable to limit our section within the bounds prescribed by the conformation of the parts, without incurring difficulties of a dangerous nature during the extraction of the stone; while, on the other hand, if we exceed these bounds, we run the risk of introducing a most active source of inflammation into the midst of the pelvis. Thus we occasionally find, on examination, that our incision has been carried fairly and completely through the prostate and neck of the bladder into the adjoining cellular tissue; allowing the entrance of the urine, and giving rise to inflammation which commences in the above structures, and spreads throughout the pelvis to the rectum and peritoneum. Again, we sometimes find the left lobe of the prostate but imperfectly divided, while the surfaces of the section are contused, lacerated, softened, and gangrenous. Here also the inflammation will be seen to commence at the wound, and subsequently extend to the pelvic cellular membrane, the rectum, and peritoneum. In both instances, the destructive process originates from the wound itself, but it is produced in each by a different cause. In the one, the knife had at once been carried into those structures where the mischief is so readily set up; in the other, the opening had been too small to allow of the removal of the stone without having recourse to dangerous violence.

Our own experience and observation lead us to concur in the statement of Baron Dupuytren, that the immediate cause of death may in most instances be ascribed to an inflammatory and suppurative process, commencing in the cellular tissue around the prostate and neck of the bladder, and extending subsequently to the peritoneum and adjacent structures; but we must be allowed to differ from him as regards the more remote cause of this inflammation, the *fons et origo* which he so strongly refers to the wound itself. At all events, we doubt the correctness of his views as applied to the mode of performing the lateral operation in England, and the principle on which it is conducted. Thus, if the section of the prostate be dexterously performed,—if the knife be carried through that body, with an obliquity outwards and backwards, the sub-peritoneal cellular membrane, which is that most susceptible of injury and morbid impressions, is never entered at all, let the incision be prolonged to ever so great an extent. The bladder is opened altogether outside the fascia, which, after lining the walls of the pelvis, becomes connected to the side of the neck of the bladder and prostate gland, and which thus constitutes a septum between the interior of the pelvis, and the lower outlet of that cavity; a septum which, if the operation be properly conducted, is rendered available for the purpose of excluding our incision from the loose, sub-peritoneal, pelvic tissue, and making it communicate only with the external wound. Under these circumstances, no parts are cut into which are very acutely sensible to the contact of the urine; and, furthermore, the hemorrhage from the divided vessels almost immediately infiltrates the surfaces of the wound, and generally prevents the entrance of the acrid fluid into the cellular membrane.

Neither do we think that M. Dupuytren is more fortunate in his second proposition, where he ascribes a similar train of inflammatory actions to the lacerated and bruised condition of the prostate gland, and commencing from the injured surface itself. It is the dragging forwards of the prostate gland before the forceps, during the unavailing efforts to extract the stone through too small an opening; it is the tearing it away from its connexions to the pubis and rectum, and not the injury to its substance, which produces the subsequent inflammation and suppuration around that body.

Our limits will not allow us to pursue this subject farther; but in whatever structure we may be inclined to place the original source of mischief, the object of the Baron is evidently to trace it to one cause, viz. the principle of the lateral operation itself; in the performance of which, he considers it impossible to make an ample and sufficient incision, unless we invade structures the lesion of which will endanger the life of the patient. These arguments may be valid in France; it is most probable that they are so; but we are quite certain that the same objections do not apply to the lateral operation as it is performed in this country, more especially where the section of the bladder and prostate is made by the knife which was first introduced by Cheselden, subsequently adopted and improved upon by Mr. Key, and now commonly employed by the majority of British surgeons. The instrument which in France is almost universally adopted for the purpose of opening the bladder is the single *lithotome caché*, which is totally inadequate to effect a very free incision. The blade is also made to assume a nearly horizontal direction, in order that the safety of the rectum may be ensured: for this instrument is by no means susceptible of the delicate manipulations which may be practised with the knife in the hands of a skilful operator. A double disadvantage is thus incurred. In the first place, the incision is limited by the ramus of the pubic arch; and, in the second, the horizontal section of the prostate, if carried through that organ, will trench upon parts which it is dangerous to disturb. This is precisely the dilemma of which M. Dupuytren complains, and to remedy which he has suggested and adopted the "Nouvelle Méthode;" but it appears to us that his objections urged against the old operation do not at all impugn its principle, although they certainly militate against the mode of executing it in his country. We believe that our French neighbours are hardly aware of the latitude of incision allowed by the use of a simple long-bladed knife, which, under the guidance of a dexterous surgeon, may be carried down by the side of the rectum, sweeping, as it were, round the intestine and producing a section which it is impossible to accomplish with the single-bladed lithotome caché. The length of the incision is not limited by the ramus of the ischium, because the direction given to the instrument is such, that it would clear the bone, even were it to be carried backwards as far as the sacro-sciatic ligaments. The incision approaches more to the vertical, stretching longitudinally instead of horizontally, and, although it is bounded on one side by the ramus of the ischium and pubis, yet on the other are situated the soft parts of the perineum and the rectum, which will readily accommodate themselves to the passage of the stone, without fear of laceration. Another facility for the extraction of the calculus is also secured by the very free division produced through the deep perineal fascia; an advantage which is not obtained even by the bilateral operation.

In what then do the advantages of the *nouvelle méthode* consist when we compare it with the lateral operation as practised in this country? They appear to be simply these:—1. It enables the operator to elude the larger vessels of the perineum.* 2. It avoids

* In the subjoined table, out of the nineteen fatal cases, two patients will be found to have sunk from hemorrhage; but, as no statement is made of the precise vessels which the necessity of laying open so great an extent of the urethra, since the canal is cut into much nearer to the bladder. 3. By including both sides of the prostate gland, it allows of a more extensive incision through that organ without completely dividing it, and consequently without implicating the surrounding plexus of veins, which sometimes prove a fruitful source of hemorrhage. 4. It secures the ejaculatory ducts from all liability to injury.

On the other hand, it must be borne in mind that the opening into the bladder is necessarily limited to the width of the prostate gland, and that by no means at its broadest part. It must also be remembered that the transgression of these bounds involves a much greater risk of mischief than can be incurred by the oblique section of the prostate. If we take into consideration the more superficial part of the wound, we shall find that the perineal fascia will be but imperfectly divided, and that the incision, being transverse, must of course be limited by the rami of the ischia on either side.

One word on the danger of cutting the rectum during the operation of lithotomy. We believe that this is a circumstance of rare occurrence in this country, although, from the repeated allusions made to it by the Baron, from the care and caution which he recommends, and the rules which he lays down to avoid such an accident, we presume it not so unfrequently happens abroad. Now, at what step of the operation is the rectum in most danger of being wounded? Certainly not during the section of the prostate gland and neck of the bladder. It is during the division of the muscles and fasciæ of the perineum, while seeking for the groove of the staff, and before the urethra is opened, that the integrity of the intestine is likely to become compromised; or it may possibly incur a risk at a subsequent period, when the knife has been introduced a second time into the bladder, for the purpose of enlarging an opening which was found too small for the extraction of the stone. A competent knowledge of anatomy, and a certain degree of manual dexterity, will, we believe, in either case, ensure the gut from all liability to injury; but, if any danger is to be apprehended in the former instance, or during the process of exposing and opening the urethra, we cannot doubt that such a danger is considerably augmented by the adoption of the transverse bilateral section, which brings the knife so much nearer to the anterior surface of the rectum than in the performance of the common lateral operation. The minute directions afforded by the Baron on this subject during the steps of the first incision, is the best proof of the correctness of our opinion.

Upon taking an impartial view of the subject in all its bearings, we do not see that the bilateral method is at all calculated to obviate were wounded, it is difficult to say whether this was an accidental untoward occurrence, or whether the divided arteries were those which run the risk of injury and occasionally produce the same fatal result after the lateral operation.—Rev.

those difficulties, those untoward circumstances, which occasionally present themselves, and which render the operation tedious and dangerous: we mean the presence of large calculi, a peculiar conformation of pelvis, an unusual depth of perineum, or a diseased and irritable condition of the urinary organs.

When this memoir was first put into our hands, we eagerly perused it in the hope of finding what has long been a desideratum among lithotomists, viz. a mode of operating which should enable us to triumph over cases of unusual difficulty and embarrassment. We shall leave our readers to draw their own inference as to how far such an object has been attained, and shall merely subjoin for their inspection the statement respecting the apparent cause of death in those who sunk after undergoing the bilateral operation. We copy the report verbatim from the table already referred to. This table contains eighty-nine cases: four of these were females, all of whom recovered. Of the remaining eighty-five males, nineteen died, making an average mortality of one in four and a half.

"Among the nineteen fatal cases just referred to, death was apparently occasioned

| | | |
|---|---|---|
| From cancer of the bladder, in . . . | | 1 case; |
| From the trilobular form of the bladder, which retained several calculi in one of its pouches, in . | | 1 |
| From a calculous affection of the prostate . . | | 1 |
| From the result of difficulties, depending | | |
| 1st. On the narrowness of the perineum . | 1 | } 4 |
| 2d. On the excessive depth of the perineum | 1 | |
| 3d. On the volume of the stones . . | 2 | |
| From subsequent spasm and delirium . . | | 1 |
| From gastro-enteritis . . . . | | 1 |
| From laceration of the prostate . . . | | 1 |
| From hemorrhage . . . . | | 2 |
| | | 12 |

"In the remaining seven cases,—that is to say, in seven cases only out of eighty-nine operations,—the patients sunk under inflammation of the bladder and the cellular tissue; produced merely by the effect of the operations, which in themselves were not accompanied with any unusual circumstances." (P. 32.)

We cannot, in justice to the editors and publishers of this work, pass over, without one word of commendation, the beautiful and indeed magnificent style in which it is produced. The plates are admirable specimens of art, and show the very high pitch to which lithographic engraving has been carried. This is one of the books of which the Brussels press may give us the letterpress, but cannot, we conceive, reproduce either the splendour or the elegance.

